# Insights into the immunomodulatory regulation of matrix metalloproteinase at the maternal-fetal interface during early pregnancy and pregnancy-related diseases

**DOI:** 10.3389/fimmu.2022.1067661

**Published:** 2023-01-09

**Authors:** Mengyu Jing, Xi Chen, Hongxia Qiu, Weihua He, Ying Zhou, Dan Li, Dimin Wang, Yonghui Jiao, Aixia Liu

**Affiliations:** ^1^ Department of Reproductive Endocrinology, Women’s Hospital, Zhejiang University School of Medicine, Hangzhou, China; ^2^ Key Laboratory of reproductive Genetics, Ministry of Education, Zhejiang University, Hangzhou, China; ^3^ Department of Obstetrics, Hangzhou Fuyang Women And Children Hospital, Fuyang, China; ^4^ Department of Obstetrics and Gynecology, First Affiliated Hospital, Zhejiang University College of Medicine, Hangzhou, China; ^5^ Department of Reproduction, People’s Hospital of Xinjiang Uygur Autonomous Region, Urumqi, Xinjiang, China

**Keywords:** matrix metalloproteinase, maternal-fetal interface immune microenvironment, pregnancy-related diseases, matrix metalloproteinase inhibitor, immunotherapy

## Abstract

Trophoblast immune cell interactions are central events in the immune microenvironment at the maternal-fetal interface. Their abnormalities are potential causes of various pregnancy complications, including pre-eclampsia and recurrent spontaneous abortion. Matrix metalloproteinase (MMP) is highly homologous, zinc(II)-containing metalloproteinase involved in altered uterine hemodynamics, closely associated with uterine vascular remodeling. However, the interactions between MMP and the immune microenvironment remain unclear. Here we discuss the key roles and potential interplay of MMP with the immune microenvironment in the embryo implantation process and pregnancy-related diseases, which may contribute to understanding the establishment and maintenance of normal pregnancy and providing new therapeutic strategies. Recent studies have shown that several tissue inhibitors of metalloproteinases (TIMPs) effectively prevent invasive vascular disease by modulating the activity of MMP. We summarize the main findings of these studies and suggest the possibility of TIMPs as emerging biomarkers and potential therapeutic targets for a range of complications induced by abnormalities in the immune microenvironment at the maternal-fetal interface. MMP and TIMPs are promising targets for developing new immunotherapies to treat pregnancy-related diseases caused by immune imbalance.

## 1 Introduction

Matrix metalloproteinase (MMP) belongs to a family of zinc(II)-dependent endopeptidases, which cleave extracellular matrix (ECM) proteins and participate in processes such as tissue remodeling and angiogenesis. From the immunological point of view, a successful pregnancy is one in which the maternal immune system can accept an embryo containing paternal antigens. Alterations in the immune environment at the maternal-fetal interface can induce immune imbalance, which can be accompanied by varying degrees of inflammatory responses, thus triggering pathological pregnancy outcomes. Tissue inhibitor of matrix metalloproteinase (TIMP), the predominant endogenous inhibitor of MMP, acts by binding MMP in a 1:1 ratio. In this review, we will provide a comprehensive overview of the immune role of MMP in the embryo implantation process and pregnancy-related diseases using data reported in PubMed and other scientific databases. Additionally, we present the possibility and potential benefits of MMP and its inhibitor as a biomarker and potential therapeutic target for pregnancy-related diseases.

## 2 Structure, functions, and regulations of MMP

### 2.1 Structure of MMP

MMP is a family of zinc(II)-dependent protein hydrolases with a common core structure that degrade the ECM, which is essential for vascular remodeling (1). Notably, zinc(II) of MMP-3 maintains its protease activity even when replaced by other ions (2). Twenty-eight types of MMP have been identified in vertebrates, and twenty-three of them are expressed in human tissues (3). MMP could be secreted by connective tissue, pro-inflammatory cells, and uterine placental cells, including fibroblasts, vascular smooth muscle (VSM), leukocytes, trophoblasts, etc.; A typical MMP consists of a pre-peptide sequence (80 amino acids), a catalytic metalloproteinase structural domain (170 amino acids), a variable length linker peptide or a hinge region, and a heme-binding protein structural domain (except for MMP-7, MMP-23, and MMP-26) (4, 5). *In vivo*, MMP generally exists as the inactive form of proMMP precursor, which is cleaved by various protein hydrolases (e.g., serine proteases, fibrinolytic enzymes, etc.), eventually producing active MMP to perform its functions (6).

### 2.2 Functions of MMP

Based on substrate specificity, MMP can be classified into gelatinase, collagenase, matrilysin, stromelysin, membrane-type MMP(MT-MMP), and others (4, 5). Because of the basic function of degrading ECM proteins, MMP is involved in a wide range of physiological and pathological processes in the human body. Physiologically, MMP plays a critical role in cell proliferation, migration and differentiation, tissue repair and remodulation, embryogenesis, and wound healing (7). Pathologically, MMP disorders are connected with tumor invasion and metastasis because MMP can degrade almost all protein components in ECM (8).

The degradation of ECM by MMP (mainly collagen and elastin) is the basis for the involvement of MMP in tissue damage repair. The degradation process of MMP requires the coordinated action of a Zn^2+^ active center and a water molecule (including three histidines and one glutamate), with methionine as a hydrophobic base to play a supporting role (5). During the MMP-substrate interaction, the Zn^2+^-bound water molecule launches a nucleophilic attack on the substrate, which eventually leads to its decomposition and release of water (9).

The process of tissue injury is inevitably accompanied by the development of inflammation, during which multiple inflammatory cells and mediators are included in the alteration. Recent research suggests that the ability to regenerate tissue may be independent of the inflammatory response, and thus the correlation between immunity and tissue regenerative capacity is gradually gaining widespread attention (10). In general, there may also be an underlying immune inflammatory response during tissue remodeling in MMP. Inflammatory cytokines (Interleukin(IL)1-α, IL1-β, IL-2, IL-17, C-reactive protein, Tumor necrosis factor-α (TNF-α), etc.) can be found in healing phases of chronic venous ulcers, which are believed to stimulate the production of neutrophil gelatinase-associated lipoprotein (NGAL),thus activating MMP-9 and form MMP-9/NGAL complexes to help to heal (11, 12). The endometrial remodeling process is precisely regulated in which MMP is essential (13). In all types of the endometrium, MMP-26 was found to have cyclical changes in its expression that may be associated with the endometrial tissue remodeling process (14, 15).

During the tumor growth and invasion process, MMP may be involved in key processes that disrupt the balance of growth and anti-growth factor signaling in the tumor microenvironment and tumor neovascularization (16). Non-catalytic functions targeting MMP are now an emerging researchhotspot. For example, the cytoplasmic tail of MT1-MMP canbind to Factor inhibiting hypoxia-inducible factor-1 (FIH-1) and promoting stable FIH-1-Munc18-1-interacting protein three interaction, which enhances hypoxia-inducible factor (HIF) target gene expression, thereby promoting Warburg effect and angiogenesis in a non-proteolytic manner (17).

This review focuses on the role of MMP in early pregnancy. The primary reproductive events include endometrial decidualization, uterine spiral artery remodeling, trophoblast cell invasion and differentiation, and placenta formation. MMP may be involved in uterine placental and vascular remodeling during normal pregnancy, as MMP is significant in tissue regulation remodeling (18).

### 2.3 MMP and ovarian sex hormone regulation

Apart from being expressed by cells, the expression of MMP can also be induced by various exogenous signals, such as cytokines, growth factors, hormones, and changes in cell-matrix and cell-cell interplay (7). Noticeably, during the implantation window period in early pregnancy, a large number of factors such as cytokines, adhesion molecules, and proteolytic enzymes are secreted by the endometrium under the mediation of estrogen and progesterone, which play critical roles in the identification and adhesion of the embryo and endometrium, and further regulate the process of embryo implantation. The interactions between steroid hormones and MMP need to be fully understood.

Affected by steroid hormones secreted by the ovary, the endometrium undergoes continuous cyclic exfoliation and remodeling throughout the female reproductive phase: estradiol stimulates endometrial proliferation during the proliferative phase of the menstrual cycle, while progesterone further acts on the estrogen-affected endometrium to induce its glandular secretion and stromal cell differentiation into metaphase cells (19). Endometrial MMP and TIMP expression regulations are essential for endometrial growth, rupture of circulating tissue, and pregnancy establishment, yet the mechanisms regulating the expression patterns of MMP and TIMP during the menstrual cycle have not been fully elucidated (20). Ovarian sex hormones (e.g., estrogen and progesterone) affect the expression of MMP, which in turn can coordinate with ovarian sex hormones to co-involve in the endometrial tissue remodeling and shedding process (21). Steroid hormone regulation of the MMP system includes direct or indirect regulation of gene transcription, specific changes in the expression, and action of local cytokines (20).

The relationship between estrogen and the specific expression of MMP family members remains unclear. Activator protein-1 (AP-1) transcription consists of c-Jun and c-Fosproteins, proven to be a significant regulator of multiple MMP transcription under multiple conditions (22). The promoters of most MMP genes contain AP-1 elements, and upon increased estrogen exposure, the ligand-bound estrogen receptor complex can increase the expression of the AP-1-bound transcription factors Fos and Jun (23).

Vitro research has shown that the addition of progesterone to endometrial explants or isolated stromal cells downregulates MMP expressions (24). Specifically,10^-8^m^-1^ estrogen and 10^-7^m^-1^ medroxyprogesterone acetate inhibited the pro-MMP-1 secreted by cultured human endometrial stromal cells (25). MMP-3 mRNA was remarkably curbed by estrogen and progestin medroxyprogesterone acetate (10^-8^mol/L-10^-6^mol/L) (26). An animal experiment using an estrogen-progestin subcutaneous implantation device to mimic the proliferative and secretory phases of the menstrual cycle in de-ovulatory rhesus monkeys found that all the MMP expressions were upregulated after progesterone withdrawal and spontaneously downregulated after menstruation in the absence of progesterone effects (27). The pattern of regulations of MMP by progesterone differs from that of estrogen. First, progesterone receptors can induce AP-1 activation in the absence of ligands, and the effect of progesterone receptors on AP-1 activity was shown to be cell type-specific (28). Second, progesterone can reverse AP-1 activation, and animal experiments suggest that progesterone can inhibit estrogen-induced c-fos mRNA expression (29). Finally, progesterone also indirectly affects the expressions of MMP by regulating cytokines (19), and IL-1α released from epithelial cells induces its expression in surrounding stromal cells through paracrine and autocrine amplification loops, thereby increasing the total amount of endometrial IL-1α and triggering MMP-1 expression (30). In contrast, progesterone at the stromal cells at the mRNA level inhibits IL-1α (19).

Human chorionic gonadotropin (HCG) has both local and systemic functions in early pregnancy (31). HCG can affect embryo attachment, embryo formation, trophoblast infiltration, and other pregnancy-related processes by up-regulating leukemia inhibitory factor (LIF), vascular endothelial growth factor (VEGF), MMP-9 and other factors, and it was reported that 500 IU/ml of HCG administration inhibited intrauterine insulin-like growth factor-binding protein-1 and macrophage-stimulating factor while elevating the level of LIF, VEGF and MMP-9 (32).The rapid increase of serum progesterone due to the rise of systemic HCG level in early pregnancy also has effects on MMP; as mentioned above, the inhibitory effect on MMP of progesterone may help limit trophoblast invasion in the proper range.

## 3 MMP and maternal-fetal interface events

### 3.1 Trophoblast invasion

Trophoblast invasion is a critical process in human placental formation, involving the regulation of cell adhesion and the degradation process of the ECM, which underlies the conversion of the uterine spiral arteries (33). To dilate the vasculature and provide adequate nutrition for embryonic growth, the extravillous trophoblast invades the maternal endometrium and remodels the spiral arteries, which allows sufficient uteroplacental perfusion (34, 35). Increased expression levels/activity of MMP-2 and MMP-9 can be found in the aorta of normal pregnant rats (36). It is suggested that these altered MMP-2 and MMP-9 may be associated with a series of cellular events at the maternal-fetal interface. EGF-mediated trophoblast invasion may be related to altered MMP-2 expression/activity (37). High levels of MMP-9 facilitate the degradation of the endometrial ECM and loosen intercellular junctions, thus favoring the invasion of extravillous trophoblast cells.With the highest levels of mRNA and protein during pregnancy at week 6-7, MMP-26 decreases gradually before reaching a minimum level at mid-gestation, and this is inconsistent with the spatiotemporal regulation of trophoblast invasive capacity (38, 39). Mishra (40) suggested that the extracellular matrix metalloproteinase inducer (EMMPRIN) may also affect embryonic adhesion and fusion with the tubular epithelium by influencing the expression of MMP-2 and MMP-14. EMMPRIN, serving as an MMP inducer, expresses membrane protein in the immunoglobulin superfamily with two heavily glycosylated extracellular structural domains (41, 42).

Notably, Nissi (43) et al. monitored serum concentrations of MMP-9, TIMP-1, TIMP-2, and MMP-2/TIMP-2 complexes in normal pregnant women at different gestational weeks, finding no statistically significant changes in levels during normal pregnancy. Few studies have been conducted on maternal serum MMP and TIMP during normal pregnancy, and further experimental studies with expanded sample sizes are needed in the future.

### 3.2 Angiogenesis and remodeling

Angiogenesis and remodeling are complex series of processes including recruitment, migration, proliferation, and apoptosis of vascular cells consisting of stem/progenitor cells, endothelial cells (ECs), vascular smooth muscle cells (VSMC), etc., while the ECM plays its essential role in vascular development and morphogenesis by providing matrix scaffolds, interacting with matrix receptors or providing growth factors (44). MMP regulates VSMC growth, proliferation, and migration processes critical in vascular remodeling. MMP-2 secretion is closely associated with the migration of VSMC from rat thoracic aorta cultured *in vitro*, involving the breakdown of the basement membrane and pericellular ECM, while in bovine studies, it was found that MMP-2 induces the migration of VSMC by triggering oxidized low-density lipoprotein (OxLDL)-induced activation of the sphingomyelin/ceramide pathway, which ultimately leads to smooth muscle cell (SMC) proliferation and migration, and that this MMP-2 activation process is mediated by MT1-MMP (45, 46). Additionally, animal studies demonstrate that MMP-2 mRNA is increased in the uterine artery at day 7 and day 21 of gestation in rats, which suggests its pregnancy-associated vascular remodeling role (47).

## 4 MMP and immune microenvironment at the maternal-fetal interface

The maternal-fetal interface is a critical site for the establishment and maintenance of normal pregnancy, where immune cell populations such as macrophages, T cells, natural killer cells, and Dendritic Cells (DCs) accumulate (48).

### 4.1 Macrophage

Macrophages are primary monocyte-derived intrinsic immune cells with remarkable heterogeneity and plasticity that are essential for homeostasis and host defense (49, 50). As the second most abundant population of leukocytes in the decidual cells, macrophages are actively involved in coordinating the apoptotic process during tissue remodeling, thereby preventing the release of potentially pro-inflammatory and pro-immunogenic cellular contents during secondary necrosis, current studies suggest that CD14 or CD68 can be used as immune markers to identify decidual macrophages (51, 52). The percentage of macrophages in leukocytes showed no significant change in early and mid-pregnancy, while the percentage of CD14^+^ macrophages tended to decrease significantly by late pregnancy (53).

It is currently believed that the phenotypic characteristics of macrophages reflect the local microenvironment response, including various cytokines and other mediators secreted by adventitial cells, and that during the embryonic implantation window, macrophages establish a pro-inflammatory microenvironment for embryonic implantation (54). *In vitro*, macrophages are usually classified into M1 and M2 phenotypes: pro-inflammatory M1 macrophages based on classical activation (LPS+ Interferon-γ (IFN-γ)), NFκB pathway, JAK/STAT signaling or IFN regulatory factor (IRF) induction and IL-4 or IL-4/IL-3-induced anti-inflammatory M2-type macrophages (55–57). Induced by IL-4, IL-10 and IL-13, M2-type macrophages have the function of anti-inflammatory by producing a large amount of IL-10 and TGF-β; Besides, its immune regulatory function is critical for early pregnancy maintenance (58).

It has been proven that decidual macrophages involved in ECM degrading in vascular remodeling are mediated by MMP-3 (59). Macrophages are an important source of MMP, and inflammatory factors can regulate the expression of macrophage proteases, including MMP (16, 60). For example, the expression of MMP-9 can be induced by TNF-α at the transcriptional level (61). Also, the MMP-9 promoter is subject to IL-18-mediated AP-1 and NF-kappaß-dependent activation (62). It was shown that MT1-MMP can control macrophage invasion by ECM components and cell surface molecular signaling (63, 64). Recent studies revealed that the transcriptional target phosphoinositide 3-kinase δ (PI3Kδ)-expressing MT1-MMP can dependently trigger Akt/GSK3 cascade signaling and ultimately restrict the expression of macrophage-derived pro-inflammatory mediators (44). The potential link between MMP and macrophages and their expression products before may be one of the directions for further work in the future.

### 4.2 Dendritic cell

Maternal immune cells and fetus-derived trophoblasts have bidirectional regulation interaction in early pregnancy, and polarization toward Th2 in the immune response is considered to be the key to successful pregnancy (65). Generally, DCs play a pivotal role in this process. Even though only approximately 1% of early pregnancy DCs are present in the decidual cells, they have a dual critical role as potent antigen-presenting cells mediating immune activation and immune tolerance, which is particularly important during pregnancy (48, 66–68). These paradoxical dual functions of DCs seem to depend on their different stages of differentiation: immature dendritic cells (iDC), characterized by DC-SIGN, promote T-cell tolerance, whereas CD83^+^ mature DCs function as an inducer of T-cell immunity (69–72). It should be noted that there also existed DEC205^+^ DCs in the metaphase stromal cells that were activated but still showed an immature state (73).

DCs are critical effectors of TGF-β activity, promoting both Peripheral T-regulatory cell production during TGF-β activation and inducing differentiation of pathogenic Th17 cells for T-cell tolerance (74). MMP may provide an alternative pathway for the proteolytic activation of potential TGF-β *in vivo*. An *in vitro* experiment demonstrates that MMP-9 depends on CD44 for cell surface localization in TA3 mouse breast cancer cells, activating TGF-β2 and TGF-β3, which promote cell invasion and angiogenesis (75).

### 4.3 Natural killer cell

Natural killer cells (NK cells), which account for more than 70% of metaphase leukocytes in early pregnancy, are represented by the CD56^+^CD16^-^ phenotype (48). According to the type of cytokines secreted, NK cells can be divided into NK1 (mainly secreting IFN-γ, TNF-α) and NK2 (mainly secreting IL-4, IL-5, IL-10, IL-13). However, the number of uterine NK (uNK) cells in the placenta decreases in late pregnancy (76). The complete spectrum of MMP expressed by NK cells and macrophages has not been determined, while *in vitro*, experimental studies have identified NK cells and macrophages expressing MMP-2, MMP-7, MMP-9, MMP-11, MMP-16, MMP-19, and TIMP (77). Current clinical studies suggest that protein array studies of CD56^+^uNK cells collected at 8-10 weeks of gestation indicate that uNK cells are the primary producers of angiogenic growth factors, but uNK cells collected at 12-14 weeks of pregnancy are the primary producers of cytokines (78).

With strong secretion of pro-inflammatory factors, angiogenic factors, etc., previous studies have reported a regulatory interaction of decidual NK cells (dNK cells) in trophoblast invasiveness through the secretion of IL-8 and interferon-inducible protein-10 (IP-10), which further stimulate CXCR1 and CXCR3-mediated pathways and thus exert regulatory effects (79). It has been suggested that IL-6 and IL-8 secreted by CD56^+^uNK cells and CD14^+^ macrophages are involved in uterine spiral artery remodeling either by secreting IFN-γ (80, 81).

**Figure 1:**
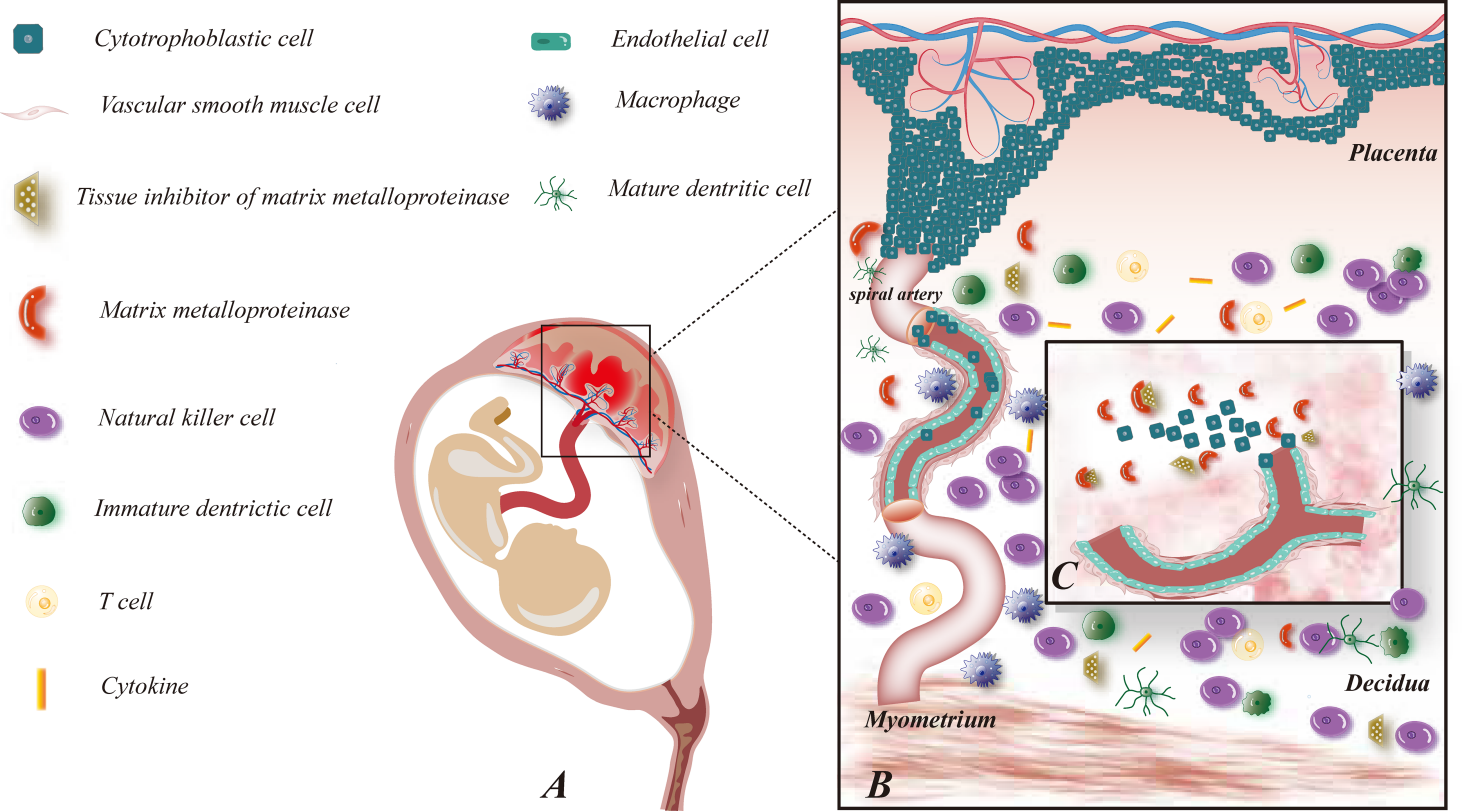
Maternal-fetal interface immune microenvironment on early pregnancy and role of MMP on embryo implantation. **(A)** This figure demonstrates the relative location of the fetus and its appendages on the maternal uterus; **(B)** This figure shows the composition of the maternal-fetal interface immune microenvironment, including fetal-originated cytotrophoblast cell, immune cell subsets: NK cells, macrophages, T cells, DC cells, cytokines, etc., maternal uterine spiral artery: vascular smooth muscle cells and vascular endothelial cells, MMP and TIMP families; **(C)** This figure illustrates the enlarged view of the functions of MMP on early pregnancy. MMP and TIMP assist the process of trophoblast invasion and uterine spiral artery remodeling by degrading ECM.

Exploration of the functions of immune cells at the maternal-fetal interface throughout pregnancy is almost impossible due to the limited access to specimens, and the speculated role of immune cells at the maternal-fetal interface in human early pregnancy is revealed in **Figure 1**. Existing studies have adequately demonstrated their essential part during pregnancy, and an in-depth understanding of their interactions can assist in identifying potential immune risks in pathological pregnancies and thus responding to them.

## 5 MMP and pregnancy-related diseases

### 5.1 Preeclampsia

Insufficient remodeling of the uterine spiral arteries is one of the necessary pathological changes in Preeclampsia (PE). The association with an acute atherosclerotic response may be one of the interactions by which inadequate spiral arterial remodeling induces placental malperfusion, in which arteries undergo an atherosclerotic process, form intimal plaques, and enter a vicious cycle of ischemia and reperfusion with an extensive intravascular inflammatory response (82, 83). Dysregulated endogenous immune responses may lead to excessive inflammatory responses, such as a significant increase in pro-inflammatory cytokines in patients with PE;At the same time, a significantly increased risk of PE in pregnant women who are in a state of inflammatory overreaction early in pregnancy (84). Abnormal immune factors inducing trophoblast under invasion and endothelial cell dysfunction are considered significant causes of PE, including innate and adaptive immune factors (85, 86). Studies have shown that pregnant women with human immunodeficiency virus infection have a lower incidence ofPEor hypertensive pregnancy (HTN-Preg), providing evidence for a strong association between organismal immunity and PE (87).

The level of MMP-2 expression in patients with PE remains controversial: some studies have found high levels of MMP-2 in the plasma or amniotic fluid of patients with PE or subsequent development of PE, while others have suggested that there is no statistical difference in the level of MMP-2 expression (88–91). However, the results of most experimental studies showed low expression or low activity of MMP-9 in serum and placental tissues of patients with PE (92). Moreover, the decrease in MMP-9 is found to be more pronounced in patients with early-onset PE (93). These findings suggest that abnormal expression of MMP-2 and MMP-9 may be involved in the pathogenesis of PE. Since MMP has a significant proteolytic effect on ECM, downregulation of MMP expression in patients with PE may lead to impaired growth, proliferation, and migration of SMC, thus interfering with the process of uterine spiral artery remodeling, which is consistent with the findings of Li (94) et al. on increased collagen content in the uterus, placenta, and aorta in mice with a model of PE. Another study showed a decrease in MMP-2 expression levels in the placenta of patients with severe PE under hypoxic conditions, mediated by the Nodal/ALK7 signaling pathway, acting in coordination with taurine upregulated protein 1 (TUG1) to achieve an invasive impaired trophoblast outcome (91).

There is extensive evidence that placental ischemia and hypoxia promote the release of a variety of active growth factors, including pro-angiogenic factors such as VEGF and placental growth factor (PlGF), as well as anti-angiogenic factors such as soluble fms-like tyrosine kinase-1 (sFlt-1), anti-inflammatory cytokines TNF-α as reactive oxygen radicals(ROS), IL-6, and HIF (18, 95). VEGF is a supergene family derived from a platelet growth factor, while sFlt-1 is a soluble antagonist of VEGF, both of which function importantly in the regulation of angiogenic homeostasis (96). An elevated sFlt-1/PIGF ratio is observed in patients with late-onset PE, and several authors have proposed sFlt-1/PIGF as an early predictor of PE (97–100). Studies support sFlt-1 as a potential upstream mechanism linking placental ischemia and reduced MMP-2 and MMP-9 content in HTN-Preg, of which VEGF can reverse this reduction in MMP content induced by sFlt-1 (94). Removal of circulating sFlt-1 in patients with early PE by a plasma-specific dextran sulfate column may reduce urinary protein and prolong pregnancy with no significant adverse fetal effects (101). sFlt-1/PIGF offers new ideas for optimizing the management of PE.

**Figure 2:**
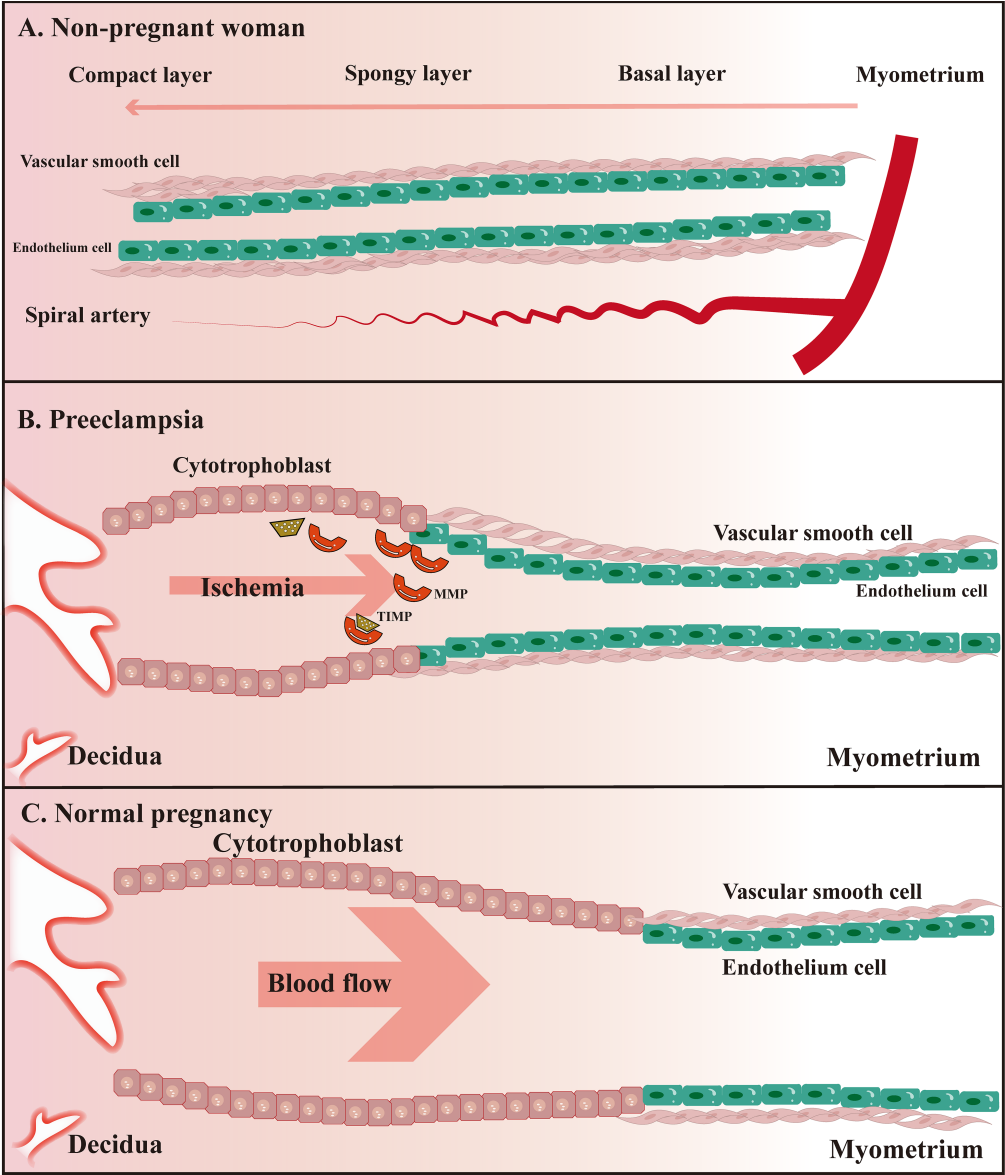
Schematic diagram of uterine spiral artery remodeling in non-pregnant women, PE, and normal pregnant women. **(A)** The uterine spiral arteries extend into the functional layer of the endometrium, of which the lumen diameter changes periodically under the influence of ovarian hormone levels; **(B)** Remodeling of the uterine spiral arteries is insufficient compared to normal pregnant women; **(C)** Physiological remodeling of the uterine spiral arteries is witnessed with the thickened spiral arteriole, enlarged lumen, and increased blood flow speed.

In addition to the abnormal remodeling process of spiral arteries, PE is also closely associated with inflammatory immune hyperactivation. Previous studies have found an excess of macrophages in placental biopsy specimens from patients with PE and that these excess macrophages tend to be located in and around the spiral arteries, separating them from trophoblast cells rather than in the stroma surrounding the spiral arteries and extravillous trophoblast (52). Apoptosis of extravillous trophoblast cells induced by macrophages through inflammatory mediators such as TNF-α is associated with damaged intravascular trophoblast invasion in PE (102). Altered MMP-9 expression in the serum of patients with PE is associated with type I TNFR, suggesting an underlying inflammatory process, especially in early PE (103). The immune mechanisms underlying the association of MMP with the development of PE are not yet clear. Here we propose the following hypothesis based on the available evidence: differential expression levels or activity of MMP (especially MMP-9) are induced by mutual promotion with various inflammatory factors (e.g., TNF-α, IL-6), causing damage to vascular ECs, and the release of ROS in hypoxic conditions to stimulate oxidative stress-inducing vascular endothelialand smooth muscle cell dysfunction (**Figure 2**).

It is also worth mentioning that MMP is involved in atherogenesis by taking an essential role in the immune response and vascular inflammation (5). The balance between synthesis and degradation of ECM components is crucial for plaque stability, and MMP, in addition to its role in degrading the ECM of patients with atherosclerosis, reflects a systemic inflammatory response whose imbalance with TIMP may be the result of changes in the environment of pro- and anti-inflammatory factors in advanced clinical stages of coronary artery disease (104). However, most of these studies were oriented towards the assessment of the role of carotid or coronary arteries, and whether MMP exerts the same effect during atherosclerosis of the uterine spiral arteries at the maternal-fetal interface in patients with PE is not yet supported by precise experimental results, which may be one of the directions worthy of future research.

### 5.2 Recurrent spontaneous abortion

Recurrent spontaneous abortion (RSA) is a severe reproductive disorder of pregnancy that remains an incomplete problem in obstetrics and gynecology (105). In addition to the removal of the N-terminal structural domain by hydrolases and regulation by endogenous inhibitors, the regulation of MMP at the gene transcription level is influenced by various cytokines and growth factors, which may closely related to abortion.

VEGF plays a vital role in embryo implantation by participating in placental development and improving endometrial tolerance. It acts mainly through binding to tyrosine kinase receptors, including Flt-1, kinase insert domain receptor (KDR, also termed VEFGR-2), Flt-4 (also termed VEGFR-3), with Flt-1 also being expressed in macrophage cell lineage cells (106–108). Lash (109) explored changes in the expression of VEGF and its receptors in the endometrium of women possessing a history of recurrent miscarriage and proposed that the expression level of VEGF-A was decreased. In another study,He and Chen (110) detected the amount of VEGF protein in the early chorionic villous tissue of patients with recurrent miscarriage by western blot and found that the amount of VEGF protein was downregulated in the early chorionic villous tissue of patients with recurrent miscarriage (0.79 ± 0.40) compared to the control group (1.01 ± 0.37). The association of the ERK-VEGF/MMP-9 signaling pathway with the epithelial-mesenchymal transitionprocess can be observed in primary hepatocellular carcinoma cells (111, 112). Indeed, the underlying cause of VEGF dysregulation in recurrent miscarriage remains unknown, and genetic variation may be one of the potential causes (113). Yan, Fang (33) showed that the MMP2-735T allele and the MMP9-1562T allele might be associated with RSA risk. Among them, the MMP9-1562T allele was also associated with preterm birth (114). Due to the critical role of VEGF and MMP in the placental implantation process, in an analysis of blood and follicular fluid from women with multiple implantation failures,Benkhalifa (115) suggested that circulating MMP-7 and VEGF could serve as potential predictive biomarkers for recurrent implantation failure.

IFN-γ is a soluble dimeric cytokine,which are higher in the peripheral blood of non-pregnant women with recurrent miscarriage than in the healthy population, suggesting IFN-γ as a potential risk factor for patients with RSA (116, 117). This is consistent with the finding that IFN-γ reduces MMP-2 secretion and trophoblast invasiveness (118). Recognizing the relationship between maternal Th1/Th2 cytokines and unexplained recurrent spontaneous abortion (URSA) helps in the early diagnosis of URSA as well as treatment monitoring, and IFN-γ/IL-4 in the early diagnosis of URSA reduces the rate of missed diagnoses (119, 120). In addition to IL-4, IL-6, IL-1, and IL-12 can be found at altered levels in patients with RSA (121–123) (**Table 1**).

**Table 1:** Interleukin levels in normal human pregnancy and recurrent spontaneous abortion.

Interleukin(IL)	Specimen	Number(n)	Normal Pregnancy (pg/ml)	RSA (pg/ml)	Trend	Method	Reference
IL-1β	Placenta	15:15	1.00*	53.58*	↑	qPCR	(123)
IL-2	Peripheral blood mononuclear cell	32:21	378.6 ± 64.9	1829.4 ± 514	↑	ELISA	(124)
IL-4	Serum	135:135	22.72 ± 15.34	8.76 ± 2.60	↓	ELISA	(125)
IL-6	Decidua	40:35	–	–	↑	RT-PCR	(126)
IL-6	Serum	40:60	0.6 ± 0.2	6.7 ± 0.9	↑	ELISA	(127, 128)
IL-10	Serum	25:24	307.7 ± 188.6	144.0 ± 106.5	↓	ELISA	(117, 129)
IL-12	Serum	18:29	8.00	12.40	↑	ELISA	(121)
IL-15	Placenta	15:12	1.00*	31.70*	↑	qRT-PCR	(130)
IL-17	Serum	40:60	1.5 ± 0.1	62.7 ± 7.8	↑	ELISA	(127)
IL-18	Placenta	15:15	1.00*	4.90*	↑	qPCR	(131)
IL-22	Decidua	11:9	5.1(3.1-5.8)**	2.9(2.0-4.4)**	↓	qRT-PCR	(132)
IL-25	Trophoblast cell	20:11	3.85(3.6-4.51)^#^	5.18(4.46-5.76)^#^	↓	qPCR	(133)
IL-27	Decidua	18:16	–	–	↓	qRT-PCR	(134)
IL-33	Decidua	6:6	–	–	↓	qPCR	(135)
IL-35	Serum	40:60	89.36 ± 33.5	54.48 ± 3.1	↓	ELISA	(127)

Values represent means ± standard deviation. Numbers shows n(normal pregnancy):n(recurrent spontaneous abortion).

* means the concentration multiplier of RSA based on normal pregnancy, which is considered to be base 1.

** means the results are calculated in ΔΔCt, and ^#^ in ΔCt. The range of quartiles is shown in parentheses.

### 5.3 Trophoblastic disease of pregnancy

Gestational trophoblastic disease (GTD) is caused by allogeneic embryo transfer and includes a series of interrelated disorders, staphyloma, invasive staphyloma, choriocarcinoma, placental site trophoblastic tumors, and epithelioid trophoblastic tumors (136). Gestational trophoblastic diseases are characterized by vascular abnormalities in the trophectoderm with an imbalance in the expression of MMP and its inhibitors (137).

Several clinical studies have shown that the imbalance between activation and inhibition of MMP-2 plays an important role in the pathogenesis, progression, and metastasis of GTD and that MMP-2 is predominantly expressed in the syncytial trophoblast of gravida (138), as well as a higher positive rate for MMP-2 and TIMP-2 in gestational trophoblastic tumors compared to normal villi (139). In addition, those with malignant potential had higher MMP-9/TIMP-1 ratios than those without malignant transformation and normal villous tissues, illustrating its potential as a predictor (140). In an *in vitro* study using 0,5,10,25,50,100,200 μg/L IL-12 to treat human choriocarcinoma cell line JEG-3, it was observed that the overall expression level of MMP-9 was reduced by IL-12 treatment compared to the control, but increased with increasing IL-12 concentration, while application of 5 μg/L IL-12 observed that MMP-9 expression was downregulated with time (0,24,36,48,72 hours) (141). IL-12 coordinates the involvement of MMP-9 in cell invasion in a dose- and time-dependent manner and is one of the possible mechanisms of choriocarcinoma development.

MMP may be involved in the invasive and metastatic potential of choriocarcinoma, which has high expression of MMPand low expression of its inhibitors. Compared to choriocarcinoma, placental site trophoblastic tumor has low expression of MMP and increased expression of inhibitors of MMP, which explains the lower invasiveness of placental site trophoblastic tumor compared to choriocarcinoma (142).

## 6 Therapeutic potential of MMP and inhibitor in pregnancy-related diseases

The role of MMP in the maternal-fetal interface makes it a promising target for immunotherapy. Overall, MMP is inhibited by both endogenous and exogenous inhibitors, with TIMP acting as the predominant endogenous inhibitor of MMP (143).

TIMP N-terminus folds as a single unit with TIMP attached to the active sites of MMP to inhibit its functions, and TIMP-1, TIMP-2, TIMP-3, and TIMP-4 homologous TIMP have been identified, of which TIMP-1 and TIMP-3 are glycoproteins (144). Intriguingly, in general, a single TIMP can inhibit multiple MMP with different effects, e.g., TIMP-1 can act simultaneously on MT1-MMP, MT3-MMP, MT5-MMP, and MMP-19 (145). Furthermore, TIMP-3 can even inhibit metalloproteinases other than MMP (146). The inability to specifically target specific MMP may be one of the reasons for the multiple side effects seen in clinical trial studies related to TIMP. This problem seems to be solved by monoclonal antibodies with high specificity and affinity for specific MMP, such as monoclonal antibodies REGA-3G12 and REGA-2D9 that react specifically with MMP-9 without cross-reacting with MMP-2 (5).

Currently, there are extensive mechanistic studies and preliminary clinical attempts regarding MMP and its inhibitor in intestinal inflammatory diseases, vascular diseases, fibrotic lesions, and tumor-related diseases. Serum MMP-3 and MMP-9 levels have been considered good markers of ulcerative colitis (UC) and inflammatory bowel disease (IBD) associated with some clinical stages (147–149). The selective MMP inhibitor ND-322 in a melanoma orthotopic mouse model can inhibit tumor growth and metastatic processes by targeting MMP-2 and MT1-MMP, providing a new avenue for adjuvant treatment options for aggressive melanoma (150). In addition, EMMPRIN is an attractive target in the treatment of oncological diseases due to its pro-angiogenic and pro-metastatic properties. Walter, Simanovich (42) designed a novel epitope-specific antibody against EMMPRIN that inhibits the secretion of MMP-9 and VEGF, shifting the tumor microenvironment of macrophages from an anti-inflammatory microenvironment dominated by TGF-β to one that is less immunosuppressive, thus allowing stimulated macrophages to perform antibody-dependent cytotoxic effects (ADCC) and kill tumor cells. There are clinical applications for EMMPRIN antibodies, such as Licartin, which has been approved by the Chinese Food and Drug Administration as a therapeutic anti-hepatocellular carcinoma radioimmune agent that is effective in reducing recurrence of hepatocellular carcinoma and prolonging survival in patients with advanced hepatocellular carcinoma after *in situ* liver transplantation (OLT) (151). Application of 50 μM concentration of docosahexaenoic acid (DHA) in human breast cancer cell line MDA-MB-231 resulted in 80% cell growth inhibition observed, while DHA inhibited breast cancer proliferation *in vitro* mainly by blocking the Cox-2-PGE2-NF-κB cascade to achieve inhibition of MMP-2 and MMP-9 transcription (152). DHA, a typical ω3-polyunsaturated fatty acid (ω3-PUFA), is one of the important unsaturated fatty acids in the body, generally from fat-rich fish, and is now widely recommended in clinical applications for nutrient supplementation and preventing preterm birth in pregnant women (153, 154), for which the preventive function is supported by *in vitro* experiment, animal experiment and clinical study (155–157). These findings provide crucial preclinical evidence for using DHA in chemoprevention to overcome potential therapeutic options for the corresponding cancers.

In the field of female pregnancy, MMP and its inhibitors have also shown surprising promise for application. Currently, the most effective treatment for PE remains the termination of pregnancy, while the incidence of preterm births will inevitably increase. Measuring the expression levels of MMP-2 or MMP-9 in serum or amniotic fluid during pregnancy may serve as a new biomarker for predicting or monitoring PE while quantifying changes in the activity of MMP by, for example, measuring protein hydrolysis products in serum may also help in the diagnosis, condition monitoring and treatment evaluation of patients with PE. Immunotherapeutic approaches play an active role in RSA (158). Since the underlying cause of RSA is still unknown, several new therapeutic approaches have been proposed to treat RSA, including low-molecular heparin, corticosteroids, intravenous immunoglobulin, or leukapheresis, but none of them have proven their effectiveness with large-scale data to date (116). Immunotherapy regimens based on MMP may be able to give a new direction to RSA. Interestingly, patients with RSA may be associated with metabolic dysregulation, such as hyperglycemia, which can affect MMP/TIMP regulation, which may provide new evidence to support clinical glycemic regulation in RSA patients (159, 160). Exploring the expression studies of MMP and TIMP members in different gestational trophoblastic diseases can help screen potential molecular biological markers for GTD diagnosis, determine the degree of disease malignancy and prognosis, and also provide possible therapeutic targets. Selective inhibitory antibodies related to MMP could be used for future treatment of gestational trophoblastic diseases. Particular inhibitory antibodies to MMP-9 and MMP-14 have been developed and shown to be effective in inhibiting tumor growth and metastasis (161), but their clinical efficacy is currently uncertain.

Despite the significant progress now acquired in MMP inhibitor research, doxycycline is the only MMP inhibitor approved by the FDA (44). Currently, MMP inhibitor therapies have not been applied in clinical practice in obstetrics and gynecology. Until we fully understand their potential mechanisms and corresponding pharmacokinetic profiles in embryo implantation and pregnancy-related diseases, the related clinical applications of MMP inhibitor immunotherapy should be cautious.

## 7 Conclusions

MMP can be secreted by various cells and is involved in processes such as tissue remodeling and angiogenesis. This paper covers the immunomodulatory mechanisms of MMP and its inhibitors at the maternal-fetal interface. However, due to the difficulty of obtaining specimens at all stages of gestational age, most existing studies have focused on maternal-fetal interface studies at early gestation or delivery, and how MMP plays a role in mid-and late pregnancy has not been elucidated, which may be one of the future research directions. This paper offers the possibility of using MMP and TIMP as targets or clinical protocols for immunotherapy in pregnancy-related diseases. However, future challenges, such as preparation of specific targeting agents and clinical side effects beyond expectations, still need to be addressed.

## Author contributions

MJ conducted the literature search, drew the images, and completed the first draft manuscript in collaboration with XC. HQ, WH, YZ, and DL helped prepare the manuscript. DW, YJ, and AL revised and edited the final version of the manuscript. All authors contributed to the article and approved the submitted version.
